# Metformin attenuates alveolar bone destruction in mice with apical periodontitis and inhibits pro-inflammatory cytokine synthesis in lipopolysaccharide-stimulated RAW264.7 through the AMPK-mTOR-NF-κB pathway

**DOI:** 10.3389/fimmu.2025.1643676

**Published:** 2025-07-31

**Authors:** Chunmei Ren, Kento Tazawa, Nobuyuki Kawashima, Risa Ohshima, Yamato Okada, Shihan Wang, Ziniu Yu, Peifeng Han, Yujin Ohsugi, Sayaka Katagiri, Takashi Okiji

**Affiliations:** ^1^ Department of Pulp Biology and Endodontics, Graduate School of Medical and Dental Sciences, Institute of Science Tokyo (Science Tokyo), Tokyo, Japan; ^2^ Department of Endodontics, Affiliated Stomatology Hospital of Guangzhou Medical University, Guangzhou, China; ^3^ Department of Oral Biology, Graduate School of Medical and Dental Sciences, Institute of Science Tokyo (Science Tokyo), Tokyo, Japan; ^4^ Section of Oral-Systemic Health, Oral Science Center, Institute of Science Tokyo (Science Tokyo), Tokyo, Japan; ^5^ Section of Vascular Cell Biology, Joslin Diabetes Center, Harvard Medical School, Boston, MA, United States; ^6^ Department of Endodontics, The Nippon Dental University School of Life Dentistry at Tokyo, Tokyo, Japan

**Keywords:** metformin, apical periodontitis, macrophages, bone destruction, anti-inflammatory agents, mammalian target of rapamycin, adenosine monophosphate-activated protein kinase

## Abstract

**Introduction:**

Apical periodontitis, caused by bacterial infection through the root canals, is characterized by chronic inflammation and bone resorption around the root apex. Metformin, a first-line therapeutic drug for type 2 diabetes mellitus, has attracted attention for its potential anti-inflammatory properties and role in regulating bone homeostasis. The hypothesis in this study was that metformin inhibits bone destruction in apical periodontitis by suppressing macrophage-mediated inflammatory responses. The aim of this study was to evaluate the effect of systemic metformin administration on experimentally induced apical periodontitis development in an animal model and clarify the underlying anti-inflammatory mechanism of metformin in lipopolysaccharide-stimulated mouse macrophages.

**Methods:**

Evaluations on the effects of metformin on the progression of periapical lesions were conducted in experimentally induced mouse apical periodontitis *in vivo*, and its anti-inflammatory effects in lipopolysaccharide-stimulated RAW264.7 macrophages *in vitro* were analyzed.

**Results:**

Metformin significantly reduced periapical bone destruction on postoperative days 21 and 28, and decreased the number of osteoclasts on the periapical alveolar bone on postoperative day 28. It also suppressed pro-inflammatory cytokine expression and nuclear factor kappa B signaling in lipopolysaccharide-stimulated RAW264.7. RNA-sequencing data revealed the downregulation of the mammalian target of rapamycin signaling after metformin treatment, which was confirmed by the downregulation of the mammalian target of rapamycin phosphorylation by metformin. Furthermore, metformin activated adenosine monophosphate-activated protein kinase, a potent negative regulator of mammalian target of rapamycin complex 1. The suppression of inflammatory cytokine expression by metformin was abolished by compound C, a potent adenosine monophosphate-activated protein kinase inhibitor.

**Discussion:**

This study revealed that metformin suppressed inflammatory bone destruction in periapical lesions. The mechanism partially involves inhibiting the mammalian target of rapamycin/nuclear factor-kappa B signaling in macrophages through adenosine monophosphate-activated protein kinase signaling activation. Findings from this study show that metformin has therapeutic potential in inflammatory bone destruction, such as apical periodontitis.

## 1 Introduction

Apical periodontitis (AP) is a chronic inflammatory condition resulting from microbial infection within the root canal. In AP, microbial stimuli passing beyond the apical foramen induce inflammation and alveolar bone destruction in the periapical tissues (1–3), and extensive destruction of the alveolar bone around the tooth apex may jeopardize tooth retention. Traditionally, root canal treatment involving debridement followed by hermetic sealing of the infected root canal spaces is the first-choice approach to eradicate intracanal infection and prevent reinfection (4). However, novel therapeutic strategies can be developed through drug therapy, which directly regulates inflammatory bone resorption in combination with etiological treatments (5).

The hallmark of AP is the infiltration of inflammatory and immune cells, including neutrophils, lymphocytes, and macrophages (6–8). Macrophages represent a major cellular constituent of AP and are thought to play key roles in its pathogenes and development by controlling various aspects of innate immunity (9). They contribute substantially by secreting critical cytokines involved in AP development, such as interleukin-1 (IL-1), IL-6, and tumor necrosis factor-α (TNF-α) (10, 11). Macrophage response is triggered by the binding of bacterial byproducts, such as lipopolysaccharide (LPS), to Toll-like receptors and is activated primarily through the nuclear factor kappa (NF-κB) signaling cascade (5, 12), leading to the development of a series of inflammatory and bone resorptive processes involving various pro-inflammatory and bone-resorptive cytokines. Moreover, macrophages undergo polarization into either the classically activated M1 subset or the M2 subset with anti-inflammatory properties based on the local microenvironment determined by various bioactive molecules (13). In addition, the state of macrophage polarization may be linked to the disease activity of AP (14). Considering those critical involvement of macrophages in AP development, targeting their activity–such as the intracellular signaling pathway linked to their pro-inflammatory cytokine production–may offer a potential therapeutic strategy for AP (9).

Metformin (MET) is an insulin-sensitizing biguanide commonly used as a first-line medication for type 2 diabetes. MET inhibits hepatic gluconeogenesis by activating adenosine 5’-monophosphate–activated protein kinase (AMPK) signaling (15) and exerts various biological functions in addition to its anti-diabetic properties, including anti-inflammatory (16, 17) and osteogenesis-promoting effects (18). Recent studies have suggested that MET, beyond its anti-diabetic properties, exerts anti-inflammatory and bone-protective effects. In a rat model of apical periodontitis, local application of MET reduced periapical bone destruction and promoted osteoblast differentiation (19). These findings imply that MET may have therapeutic potential in AP; however, the underlying mechanisms, particularly its impact on inflammation-related pathways, remain unclear. Given the central role of macrophage-mediated inflammation in AP pathogenesis, investigating whether MET modulates macrophage inflammatory responses may help clarify its mechanism of action and therapeutic relevance in AP.

In this study, the aim was to evaluate the effect of systemic MET administration on experimentally induced AP development in an animal model and clarify the underlying anti-inflammatory mechanism of MET in LPS-stimulated mouse macrophages. The hypothesis of the study was that metformin inhibits bone destruction in apical periodontitis by suppressing macrophage-mediated inflammatory responses.

## 2 Materials and methods

### 2.1 AP animal model and drug administration

All animal experiments were conducted in accordance with the guidelines established by the Institutional Committees for Animal Experiments at Tokyo Medical and Dental University (currently the Institute of Science, Tokyo, Japan), and all experimental protocols were approved by these committees (#A2023-196C3).

**Figure 1:**
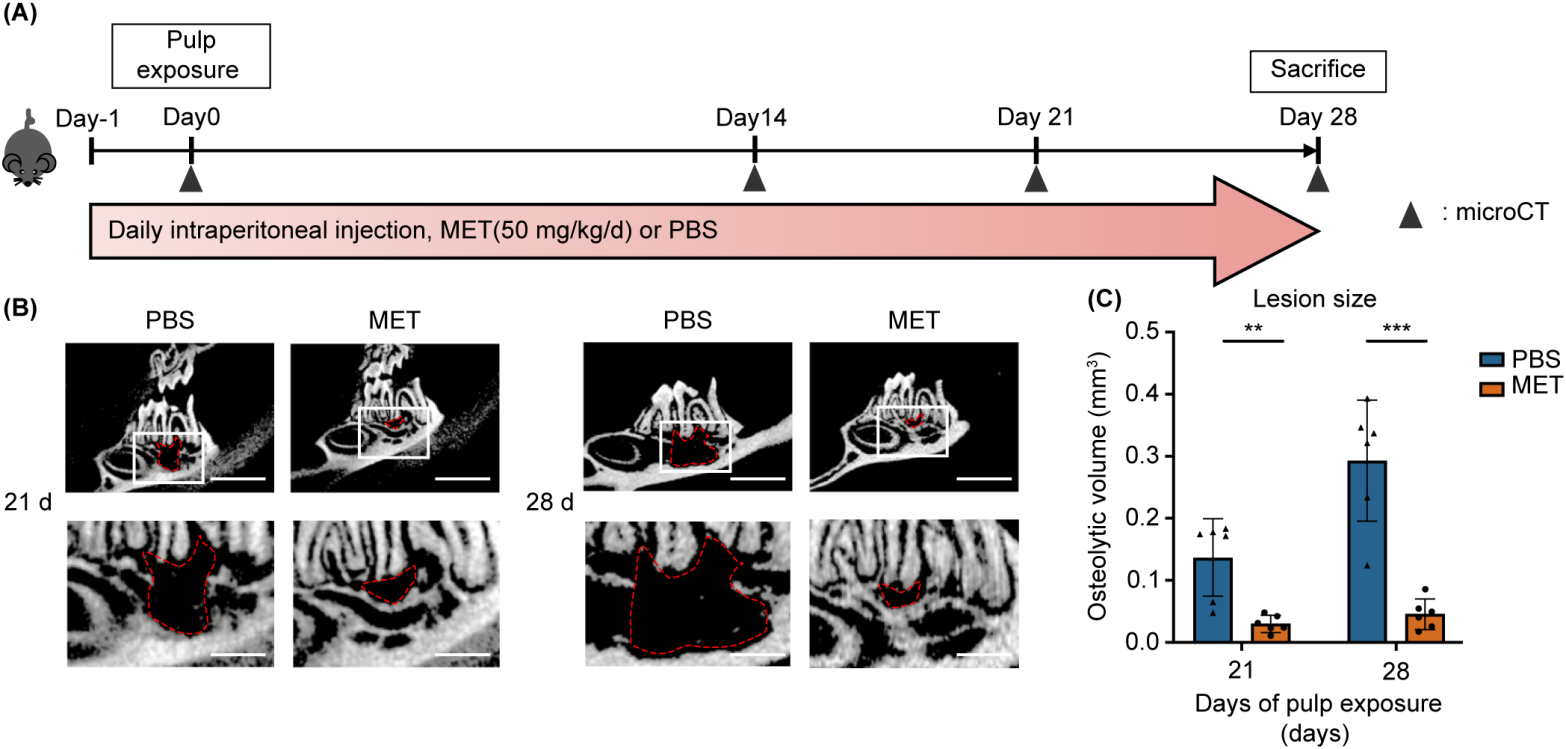
MET administration reduces experimentally induced periapical bone destruction. **(A)** Time course and treatments in *in vivo* experiments. The mice received a daily intraperitoneal injection of MET (50 mg/kg, l00 μL, n = 3) or PBS (100 μL, n = 3). The right and left mandibular first molars were subjected to pulp exposure and left open for the development of apical periodontitis. **(B, C)** Representative micro-CT images and quantitative analysis of the periapical lesion sizes on postoperative days 21 and 28. Micro-CT scans showing representative images of the periapical lesion areas of the mandibular first molars in the PBS and MET groups, with manual contour delineation of the periapical radiolucency **(B)**. MET-treated mice exhibited significantly smaller lesion sizes than did the PBS-control mice on postoperative days 21 and 28 **(C)**. Values are shown as the mean ± standard deviation. **P < 0.01, and ***P < 0.001. Scale bar = 2 mm (upper) and 6 mm (lower).

C57BL/6 JJc1 mice (male, 6 weeks old, n = 9) were obtained from CLEA Japan, Inc. (Tokyo, Japan). The protocol for inducing AP has previously been described (20). In brief, the animals were anesthetized via an intraperitoneal injection of ketamine (75 mg/kg) and dexmedetomidine (1 mg/kg) and mounted on a handmade mouth-opening apparatus. Cavity preparation was performed to expose the dental pulp on the left and right mandibular first molars using an electric handpiece (Vivamate G5, Nakanishi, Kanuma, Japan) with a 1/4 round carbide bur (#14820, SS White Dental, Lakewood, NJ, USA) under a stereomicroscope (Zeiss, Oberkochen, Germany). Subsequently, the coronal pulp was removed with a stainless steel K-file #8 (Dentsply Maillefer, Ballaigues, Switzerland) into the mesial and distal canals, and the exposure was left open. Pulp-exposed mice were randomly assigned to the MET administration and phosphate-buffered saline (PBS) control groups using a computer-generated randomization list by an independent researcher blinded to the study protocol (n = 3 in each group). Given its established safety profile and suitability for long-term administration, MET was administered intraperitoneally at a dose of 50 mg/kg/day in this study to investigate its therapeutic effects on AP. This dosage and route of administration were selected based on previous *in vivo* studies demonstrating their efficacy and relevance in evaluating the pharmacological effects of MET in rodent models (21). The mice received MET in 100 μl PBS or the same volume of PBS intraperitoneally daily for 28 days, starting 1 day before the surgery. The mandible of each mouse was scanned under anesthesia on postoperative days 7, 14, 21, and 28 using *in vivo* micro-computed tomography (micro-CT; inspeXio SMX-100CT, Shimadzu, Tokyo, Japan; **Figure 1A**) with settings of 70 kV, 140 μA, and a 9 μm voxel size. The same animals were euthanized with carbon dioxide on postoperative day 28, and the mandibles were isolated for histological evaluation.

For the negative control, non-treated mice (n = 3) were sacrificed, and their mandibles were subjected to micro-CT to assess the periodontal ligament space in healthy teeth. The volume of apical bone resorption was calculated as previously described (22). In brief, the region of interest was set as the osteolytic and periodontal spaces around the distal root of the first mandibular molar. A blinded observer manually delineated the contours of these regions using CT-analyzer software (Amira, Thermo Fisher Scientific, Waltham, MA, USA) to reconstruct the volume of the periapical lesions. Subsequently, an automated threshold script was used to measure the total volume of bone loss. The volume of true bone loss was determined by subtracting the space of the periodontal ligament in healthy teeth from the volume of total bone loss.

### 2.2 Sample preparation and histology

The mandibular samples were fixed in a 4% paraformaldehyde solution at 4°C for 24 h and decalcified with 17% ethylenediaminetetraacetic acid at 4°C for 4 weeks. The tissues were placed in 15% sucrose in PBS for 12 h, transferred to 30% sucrose in PBS until the tissues sank, and embedded in an optimal cutting temperature compound. Frozen sections (5 μm thick) were obtained using a cryostat (Leica CM3050 S, Nussloch, Germany) and subjected to hematoxylin and eosin and to tartrate-resistant acid phosphatase (TRAP) staining. TRAP staining was performed as follows: The TRAP basic incubation medium was prepared by dissolving sodium acetate anhydrous (9.2 g; S-2889, Sigma-Aldrich, Burlington, MA, USA) and L-(+)-tartaric acid (11.4 g; T-6521, Sigma-Aldrich) in 950 mL distilled water, followed by adding 2.8 mL glacial acetic acid. The pH was adjusted to 4.7–5.0 using 5 M sodium hydroxide, and the final volume was made up to 1 L using distilled water. Next, naphthol AS-MX phosphate substrate mix was prepared by dissolving naphthol AS-MX phosphate (20 mg; N-4875, Sigma-Aldrich) in ethylene glycol monoethyl ether (1 mL; E-2632, Sigma-Aldrich) followed by thorough mixing. The working TRAP staining solution was freshly prepared by combining 200 mL of TRAP basic incubation medium, Fast Red Violet LB Salt (120 mg; F-3381, Sigma-Aldrich), and 1 mL naphthol AS-MX phosphate substrate mix, followed by thorough mixing before use. After incubation with TRAP staining solution, the sections were counterstained with 0.08% Fast Green (Santa Cruz Animal Health, Dallas, TX, USA), rinsed with distilled water, air-dried, and mounted in xylene. Images were captured with a light microscope (Nikon ECLIPSE Ci, Tokyo, Japan) and subjected to quantitative analysis using image processing software (ImageJ v2; National Institutes of Health, Bethesda, MD, USA). Tissue sections were examined to identify the lesion area, specifically the periapical region surrounding the distal root of the mandibular first molar. The mandibular first molar was centered in the field of view of the microscope, and the entire periapical region was systematically scanned. TRAP-positive cells were manually counted across the entire root apex. In brief, for the alveolar bone surface, the length along the alveolar bone margin was measured in millimeters (mm), and the number of TRAP-positive cells per unit length (/mm) was quantified. The apical periodontitis lesion area was measured, and the number of TRAP-positive cells (/mm²) was calculated (n = 6 in the PBS group and 5 in the MET group).

### 2.3 Cell culture and reagents

RAW264.7 cells, a mouse macrophage cell line, were obtained from the Cell Engineering Division, RIKEN BioResource Research Center (Tsukuba, Japan; Cell No. RCB0535). The cells were cultured in Dulbecco’s modified Eagle’s medium (FUJIFILM Wako Chemicals, Tokyo, Japan) supplemented with heat-inactivated 10% fetal bovine serum (Thermo Fisher Scientific, #1600-500, Waltham, MA, USA) and 1% penicillin-streptomycin (FUJIFILM Wako Chemicals) at 37°C with 95% oxygen and 5% carbon dioxide. The following drugs and reagents were used in this study: metformin hydrochloride (138-18661: FUJIFILM Wako Pure Chemicals), lipopolysaccharide (*Escherichia coli* O111:B4; Sigma-Aldrich), compound C (171260; Merck Millipore, Burlington, MA, USA), MHY1485 (S7811; Selleck Chemicals, Houston, TX, USA), and BAY 11-7085 (196309-76-9; Cayman Chemical, Ann Arbor, MI, USA).

### 2.4 Cell treatment

RAW264.7 macrophages were plated at 2 × 10^5^ cells per well in 12-well plates and allowed to adhere overnight in DMEM. Cells were then pre-treated with MET (0.5 mM) for 6 h, followed by stimulation with LPS (100 ng/mL, 4 h). To activate mTOR, the agonist MHY1485 (MHY, 2 µM) was added for 3 h after MET treatment. To inhibit AMPK, Compound C (CC, 5 µM) was applied for 1 h following MET treatment, and the cells were subsequently stimulated with LPS (100 ng/mL, 4 h). Experimental groups therefore comprised (i) untreated control, (ii) LPS alone, (iii) MET + LPS, (iv) MET + CC + LPS, and (v) MET + MHY. The same treatment schedule was applied for all downstream assays, including RNA-seq, quantitative PCR, western blotting and luciferase reporter analysis.

### 2.5 Cell proliferation assay

The effect of MET on cell viability was determined using the Cell Counting Kit-8 (Dojindo Laboratories, Kumamoto, Japan), as previously described (23). In brief, RAW264.7 cells were plated in 96-well plates at a density of 7 × 10^3^ cells/well and incubated overnight. The cells were treated with 0, 0.5, or 1 mM of MET. Cell proliferation was measured at 0, 24, 36, and 72 h using a spectrophotometer (Sunrise, Tecan, Männedorf, Switzerland).

### 2.6 Real-time quantitative polymerase chain reaction

Total RNA was extracted using the QuickGene RNA Cultured Cell Kit S (FUJIFILM Wako Chemicals), and 300 ng/mL of RNA was subjected to cDNA synthesis using PrimeScript™ RT Master Mix (Takara Bio, Kusatsu, Japan). Real-time quantitative polymerase chain reaction (qPCR) was performed using the specific primers outlined in **Table 1** and the GoTaq qPCR Master Mix (Promega, Madison, WI). The amplification process was performed on the CFX96 Real-Time qPCR System (Bio-Rad, Kidlington, UK). The gene expression was calculated using the formula 2^−ΔΔCt^ with β-actin as an internal control.

**Table 1:** Primer sequences used in this study.

Gene	Forward (5’-3’)	Reverse (5’-3’)	Accession No.	Size (bp)
(mouse)				
*Il1a*	CACCTTACACCTACCAGAGTGATTT	ATTTAACCAAGTGGTGCTGAGATA	NM_010554	137
*Il1b*	AAACGGTTTGTCTTCAACAAGATAG	AATTATGTCCTGACCACTGTTGTTT	NM_008361	141
*Il6*	TGGATGCTACCAAACTGGATATAAT	TCTGGCTTTGTCTTTCTTGTTATCT	NM_031168	130
*Tnfa*	GATGGGTTGTACCTTGTCTACTCC	GAGGTTGACTTTCTCCTGGTATGAG	NM_013693	120
*Mcp1*	GAGAAAGCTGAGTTGACTCCTACTG	TTTCTGAGGTAGGTTCTTTCTCTCC	NM_008570.1	130
*β-actin*	AATGATCTTGATCTTCATGGTGCTA	GTAAAGACCTCTATGCCAACACAGT	NM_007393	122

*Il1a*, interleukin 1 alpha; *Il1b*, interleukin 1 beta; *Mcp1*, monocyte chemotactic protein 1; *Tnfa*, tumor necrosis factor alpha; *β-actin*, beta-actin.

### 2.7 RNA-sequencing analysis

RNA sequencing was performed by Rhelixa Co. (Tokyo, Japan). Quality control and alignment procedures were applied to FASTQ data to generate an expression matrix. This process entailed using fastp software on a Linux server for data quality control, followed by alignment of the FASTQ reads to the mouse mm10 reference genome using hisat2 software. Quantification and normalization were performed using feature counts and DESeq2, respectively. Principal component analysis was conducted and visualized using the prcomp function in the R package. The plots and graphs generated from the RNA-sequencing analysis were visualized using the R software, version 4.3.3 (R-Project, Vienna, Austria). Gene set enrichment analysis (http://software.broadinstitute.org/gsea/index.jsp) was performed using the hallmark gene sets.

### 2.8 Western blotting

The cells were lysed using radioimmunoprecipitation assay buffer (Merck Millipore) supplemented with protease and phosphatase inhibitor cocktails (cOmplete and PhosSTOP; Sigma-Aldrich) at 4°C for 15 min. Protein samples were heated to boiling temperature for 5 min in 1× sodium dodecyl sulfate buffer lysates. The proteins were separated by 10–20% e-PAGEL (ATTO, Tokyo, Japan) and transferred onto a polyvinylidene difluoride membrane (Merck Millipore) by semi-dry transfer at 0.15 mA for 1 h using a blotting system (WSE-4040, ATTO). The antibodies used in this study included rabbit monoclonal antibodies (Cell Signaling Technology, Danvers, MA) against p65/RELA (1:1,000; D14E12), phospho-NF-κB p65 (1:1,000; 93H1), phospho-AMPKα (Thr172) (1:2000; D79.5E), AMPK (1:1,000; D5A2), mTOR (1:1,000; 7C10, Cell Signaling Technology), and p-mTOR (1:1,000; D9C2),. Horseradish peroxidase-conjugated anti-glyceraldehyde-3-phosphate dehydrogenase (1:4,000; M171-7, MBL Life Science, Tokyo, Japan) and horseradish peroxidase-labeled anti-rabbit IgG (1:5000, W4011, Promega) were also used. After applying a chemiluminescent substrate (Immobilon, Merck Millipore), protein bands were detected using the LAS-3000 mini-imaging system (FUJIFILM Wako Chemicals). Protein expression levels were measured using the ImageJ software v2 (National Institutes of Health).

### 2.9 Luciferase assay

The NF-κB signaling activity was evaluated using the pGL4.32 (luc2P/NF-κB-RE/Hygro, Promega) vector, which contains five copies of the NF-κB response element. RAW264.7 cells were transfected with the reporter vector using the Lipofectamine 3000 transfection reagent from Thermo Fisher Scientific. The cells were lysed using a luciferase cell culture lysis reagent (#E1483, Promega). Subsequently, the activity of the light-producing enzyme luciferase was measured using a luciferase assay reagent (#E1483, Promega) and a luminometer (Luminescence PSN, ATTO).

### 2.10 Statistical analysis

Statistical analyses were performed using the Statistical Package for Social Sciences (version 9.10; SPSS Inc., Chicago, IL, USA) and GraphPad Prism (version 9; GraphPad Software Inc., La Jolla, CA, USA). Normality distributions were assessed using the Shapiro–Wilk test, and Levene’s test was used for variance equality comparisons. For normally distributed variables with equal variance, the unpaired Student’s t-test was applied for comparisons between two groups, and a one-way analysis of variance followed by Tukey’s *post hoc* test was used for comparisons among three or more groups. For experiments involving two independent variables, a two-way analysis of variance was used to assess the main effects and interactions between factors, followed by Tukey’s multiple comparisons test when appropriate. When the normality assumption was met, but the variances were uneven, the unpaired Welch’s t-test was used for two-group comparisons, and the Brown-Forsythe/Welch one-way analysis of variance with Dunnett’s T3 *post hoc* test was used for three or more groups. For data with non-normal distribution, the Mann–Whitney U test was employed for two-group comparisons, and comparisons among three or more groups were conducted using the Kruskal–Wallis test with Dunn’s *post hoc* test. Statistical significance was set at p < 0.05.

## 3 Results

### 3.1 MET administration markedly reduced periapical bone destruction

**Figure 2:**
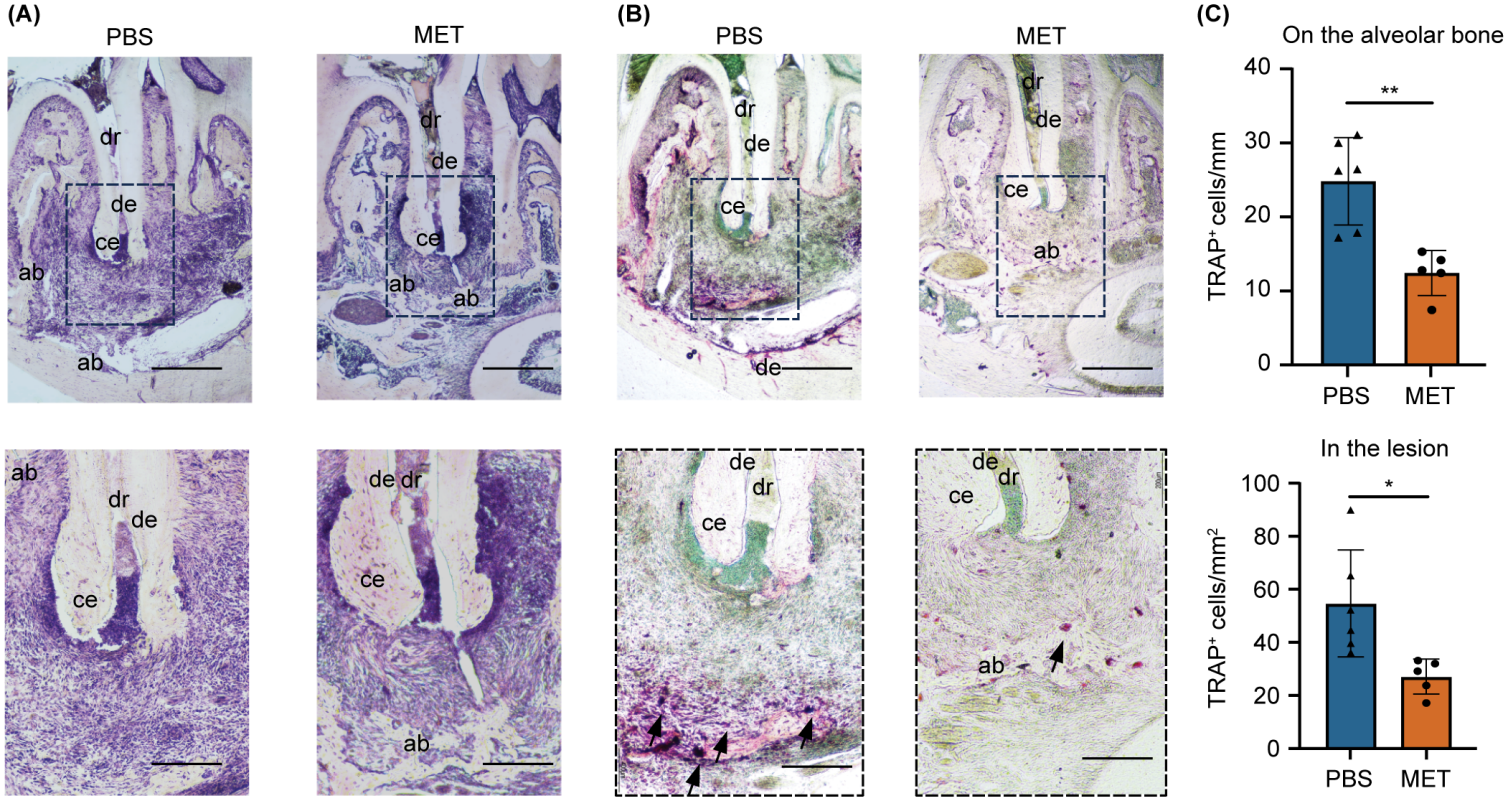
MET reduces inflammatory infiltrates and tartrate-resistant acid phosphatase (TRAP)-positive cells in experimentally induced apical periodontitis. **(A)** Representative histological images of experimentally induced apical periodontitis in mouse mandibular first molars using hematoxylin and eosin staining. The area enclosed by the dotted line is shown at high magnification. MET-treated mice (right) showed a lower degree of inflammation. **(B)** Representative tartrate-resistant acid phosphatase (TRAP)-stained images of experimentally induced apical periodontitis in mouse mandibular first molars. TRAP-positive cells were located close to the periapical bone in the PBS control group (left) but were few in the MET group (right). **(C)** Quantitative analysis of TRAP-positive cell counts. Significantly more TRAP-positive cells were detected on the alveolar bone (upper panel) and in the lesions (lower panel) of the PBS than in those of the MET groups (PBS group, n = 6; MET group, n = 5). Values are expressed as the mean ± standard deviation. *P < 0.05, and **P < 0.01. Scale bar = 400 μm (upper panels in a and b) and 200 μm (lower panels in a and b). dr, distal root; ab, alveolar bone; ce, cementum; de, dentin.

The effect of MET on AP development was evaluated using a mouse AP model induced by making unsealed surgical pulp exposure in mandibular first molars. The mice received daily intraperitoneal injections of MET, starting a day before AP induction and continuing until sacrifice (**Figure 1A**). In the control group, periapical bone loss increased progressively over time, with significant bone resorption observed on postoperative days 21 and 28 (**Supplementary Figure S1A**). In contrast, bone resorption was notably suppressed in the MET group on postoperative days 21 and 28 (**Figures 1B, C**). Histological analysis of the periapical lesions showed that inflammatory cell infiltration was suppressed in the MET group compared to the control group (**Figure 2A**). In addition, the MET group showed a significantly lower number of cells positive to TRAP on postoperative day 28 than did the control group (**Figures 2B, C**).

### 3.2 MET suppressed LPS-induced pro-inflammatory cytokine expression in RAW264.7 cells

**Figure 3:**
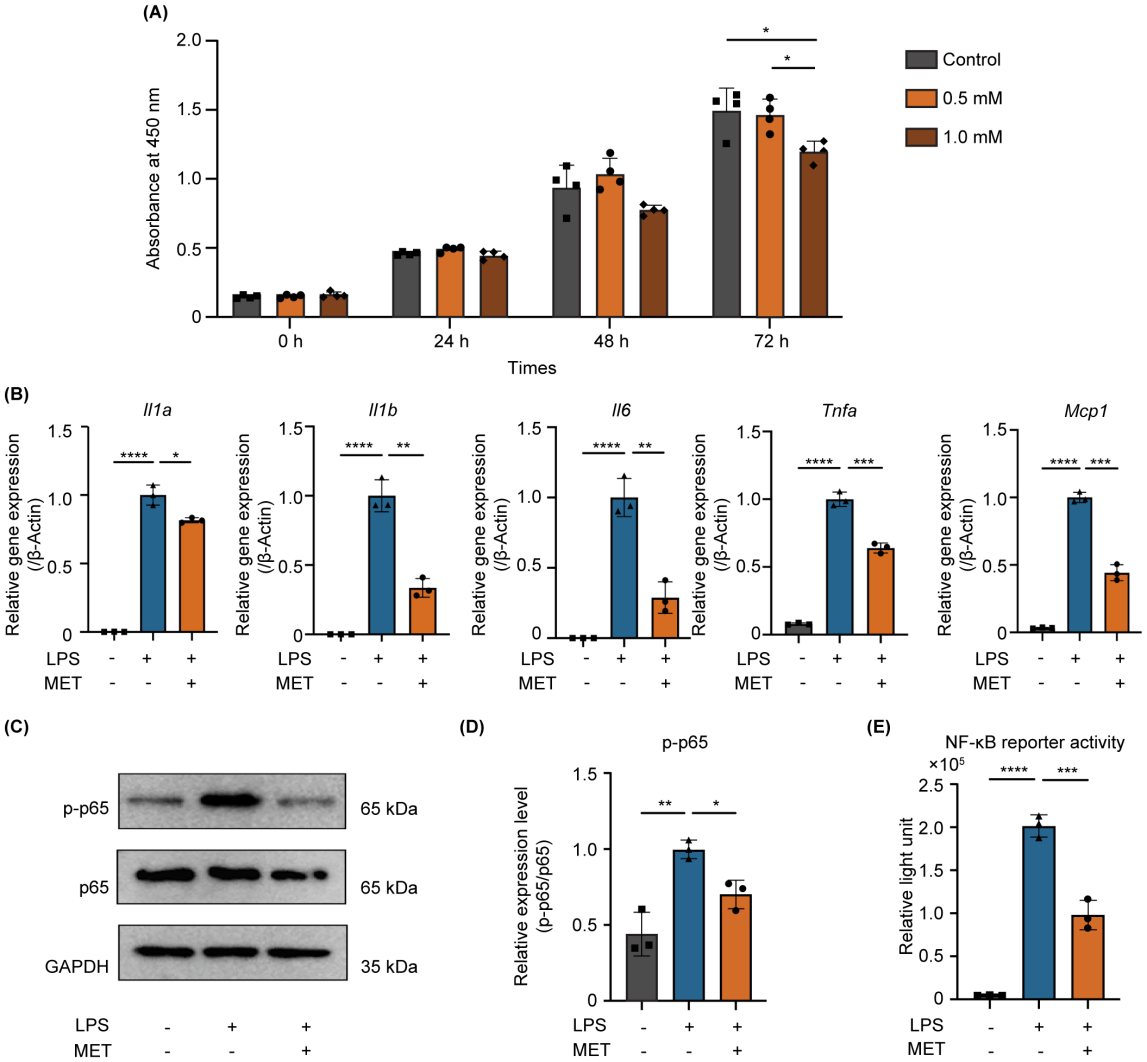
MET inhibits NF-κB signaling and suppresses pro-inflammatory cytokine expression in LPS-stimulated RAW264.7 cells. **(A)** Effect of MET on RAW264.7 cell viability. CCK8 assays showing the effect of MET on RAW264.7 cell viability at various concentrations and time points. Cell viability markedly decreased after treatment with 1.0 mM MET at 72 h (n = 4). MET (0.5 mM, for 6 h) was selected for subsequent functional studies. **(B)** Pro-inflammatory cytokine expression in LPS-stimulated RAW264.7 cells in the presence or absence of MET. The expression of pro-inflammatory cytokines (*Il1a, Il1b, Il6*, *Tnfa*, and *Mcp1*) was induced by treatment with 100 ng/mL LPS for 4 h but significantly decreased by MET treatment. **(C, D)** Effect of MET on p-p65 expression in LPS-stimulated RAW264.7 cells. LPS-induced p-p65 expression is downregulated in the presence of MET **(C)**. The p-p65/p65 ratio was significantly increased by LPS treatment, which was inhibited by MET pretreatment. **(E)** Effect of MET on NF-κB signaling activity. Luciferase assay confirmed that NF-κB activity induced by LPS was significantly decreased by MET treatment. Values are shown as the mean ± standard deviation. *P < 0.05, **P < 0.01, ***P < 0.001, and ****P < 0.0001; n = 3.

MET was applied to LPS-stimulated RAW264.7 cells to investigate the anti-inflammatory effects of MET on pro-inflammatory macrophages. Initially, the cytotoxicity of MET was assessed to determine the appropriate concentration for use. Cell viability was not affected by 0.5 mM MET. However, 1.0 mM MET notably reduced cell viability at 72 h (**Figure 3A**). Based on these results, a concentration of 0.5 mM was selected for subsequent *in vitro* experiments. Real time-quantitative polymerase chain reaction revealed that MET markedly reduced the mRNA expression of pro-inflammatory cytokines (*Il1a*, *Il1b*, *Il6*, *Tnfa*, and *Monocyte chemotactic protein1(Mcp1)*) in LPS-stimulated RAW264.7 cells (**Figure 3B**). Furthermore, phosphorylated-p65 (p-p65) expression, which peaked at 30 min in LPS-stimulated RAW264.7 cells (**Supplementary Figures S2A, B**) was markedly reduced by 0.5 mM MET (**Figures 3C, D**). LPS-induced NF-κB activity was markedly suppressed by MET treatment (**Supplementary Figure S2C**; **Figure 3E**).

### 3.3 MET downregulated mammalian target of rapamycin dependent NF-κB signaling

**Figure 4:**
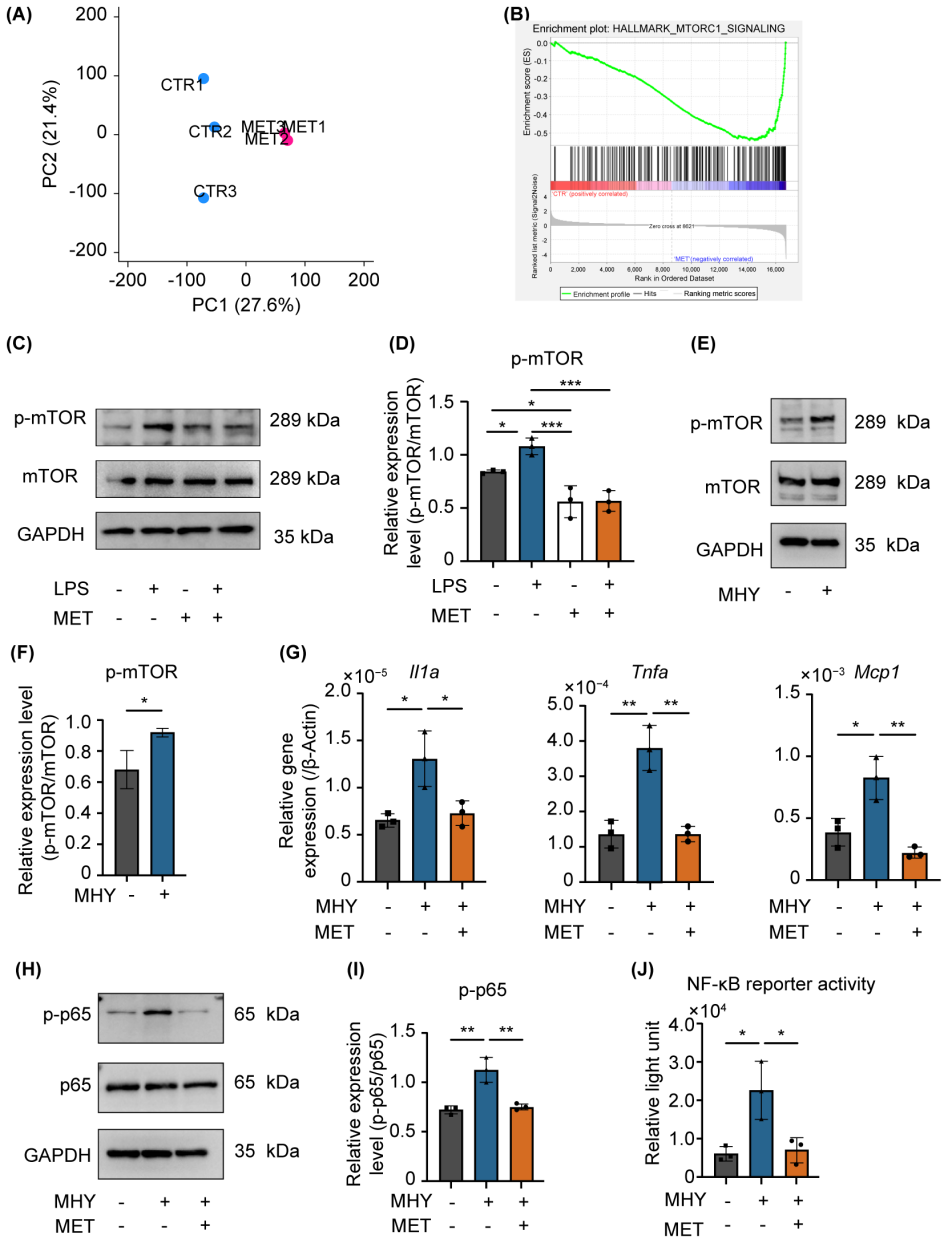
MET modulates the mannmalian target of rapamycin (mTOR) signaling induced by LPS stimulation. **(A)** Gene expression patterns determined using principal component analysis (CTR: control, MET: metformin-treated RAW264.7 cells). The expression patterns in the MET group were different from that of the CTR. **(B)** Gene set enrichment analysis of MET-treated RAW264.7 cells and control. The mTORC1 signaling gene set was downregulated in MET-treated RAW264.7 cells compared with that of the CTR. **(C, D)** Effects of MET on LPS-induced mTOR phosphorylation. A representative image of western blotting **(C)**. Quantitative analysis of protein expression level **(D)**. MET treatment significantly downregulated the protein expression of p-mTOR induced by LPS. **(E, F)** Effect of MHY, an mTOR agonist, in RAW264.7 cells. Representative western blotting image **(E)**. Quantitative analysis of protein expression level **(F)**. MHY treatment upregulated the expression of p-mTOR (2 μM for 3 h). **(G)** mRNA expression of pro-inflammatory cytokines in the presence of MET in MHY-treated RAW264.7 cells. MHY significantly upregulated the mRNA expression of pro-inflammatory cytokines *Il1a, Tnfa* and *Mcp1*, whereas MET suppressed the induction of MHY-induced pro-inflammatory cytokines. **(H, I)** Effect of MET and MHY on p-p65 expression. A representative western blotting image **(H)**. Quantitative analysis of protein expression level **(I)**. MHY upregulates p-p65 expression, which was inhibited by MET treatment. **(J)** Effect of MET on NF-κB signaling activity induced by MHY. Luciferase assay confirmed that MHY increases NF-κB activity which significantly decreased by MET treatment. Values are shown as the mean ± standard deviation. *P < 0.05, **P < 0.01, and ***P < 0.001; n = 3.

A comprehensive genetic analysis was performed on the control and MET-treated RAW264.7 cells using next-generation sequencing to elucidate the mechanism by which MET inhibits NF-κB signaling. The principal component analysis scatter plot showed a distinct gene expression profile between the control and MET-treated samples, with a 27.6% proportion of variance in principal component (PC) 1 (**Figure 4A**). Gene set enrichment analysis using hallmark gene sets was conducted to assess the differences in mRNA expression levels between the control and MET-treated RAW264.7 cells (**Figure 4B**). A false discovery rate q-value of < 0.05 is shown in the gene sets in **Table 2**. Of these, the mammalian target of rapamycin complex 1 (mTORC1), a signaling pathway associated with inflammation (24), was significantly downregulated in the MET group (*P* < 0.05), suggesting that MET exerted anti-inflammatory effects on mTOR signaling under the current experimental conditions.

**Table 2:** Gene set enrichment analysis between control and MET-treated samples.

Gene set	Size	NES	NOM *P*-Value	FDR q-Value
Downregulated after MET treatment				
Cholesterol homeostasis	63	-2.52	< 0.001	< 0.001
Tnfα signaling via NF-κB	165	-2.4	< 0.001	< 0.001
Hypoxia	145	-2.27	< 0.001	< 0.001
mTORC1 signaling	190	-2.04	< 0.001	< 0.001
P53 pathway	172	-2.03	< 0.001	< 0.001
Interferon-alpha response	85	-1.9	< 0.001	0.001
Interferon-gamma response	154	-1.83	< 0.001	0.001
Unfolded protein response	108	-1.77	< 0.001	0.002
Glycolysis	161	-1.72	< 0.001	0.004
Hedgehog signaling	20	-1.57	0.027	0.022

FDR, false discovery rate; NES, normalized enrichment score; NOM, nominal.

MET significantly reduced the expression of phosphorylated mTOR (p-mTOR) in LPS-stimulated RAW264.7 cells (**Figures 4C, D**). MHY1485 (MHY, 2 µM), an agonist of mTOR, upregulated the expression of p-mTOR (**Figures 4E, F**) and the mRNA expression of pro-inflammatory cytokines (*Il1α, Mcp1*, and *Tnfa*), which were notably downregulated by MET treatment (**Figure 4G**). Furthermore, MET markedly downregulated MHY-induced expression of p-p65 (**Figures 4H, I**) and the activity of NF-κB (**Figure 4J**). Next, the effect of BAY11-7085 (BAY, 2 µM), an inhibitor of IκBα phosphorylation was examined (25), because LPS can activate NF-κB signaling through the mTOR pathway (26). BAY11–7085 markedly reduced the mRNA expression of LPS-induced pro-inflammatory cytokines (*Il1a, Il1b*, and *Mcp1*) and NF-κB activity, which were restored by MHY (**Supplementary Figures S3A, B**). These findings indicate that MET abrogates NF-κB activation through the mTOR pathway, which is critical in driving inflammatory mediator synthesis in RAW264.7 cells stimulated with LPS.

### 3.4 AMPK activated by MET suppressed mTOR-induced NF-κB signaling

**Figure 5:**
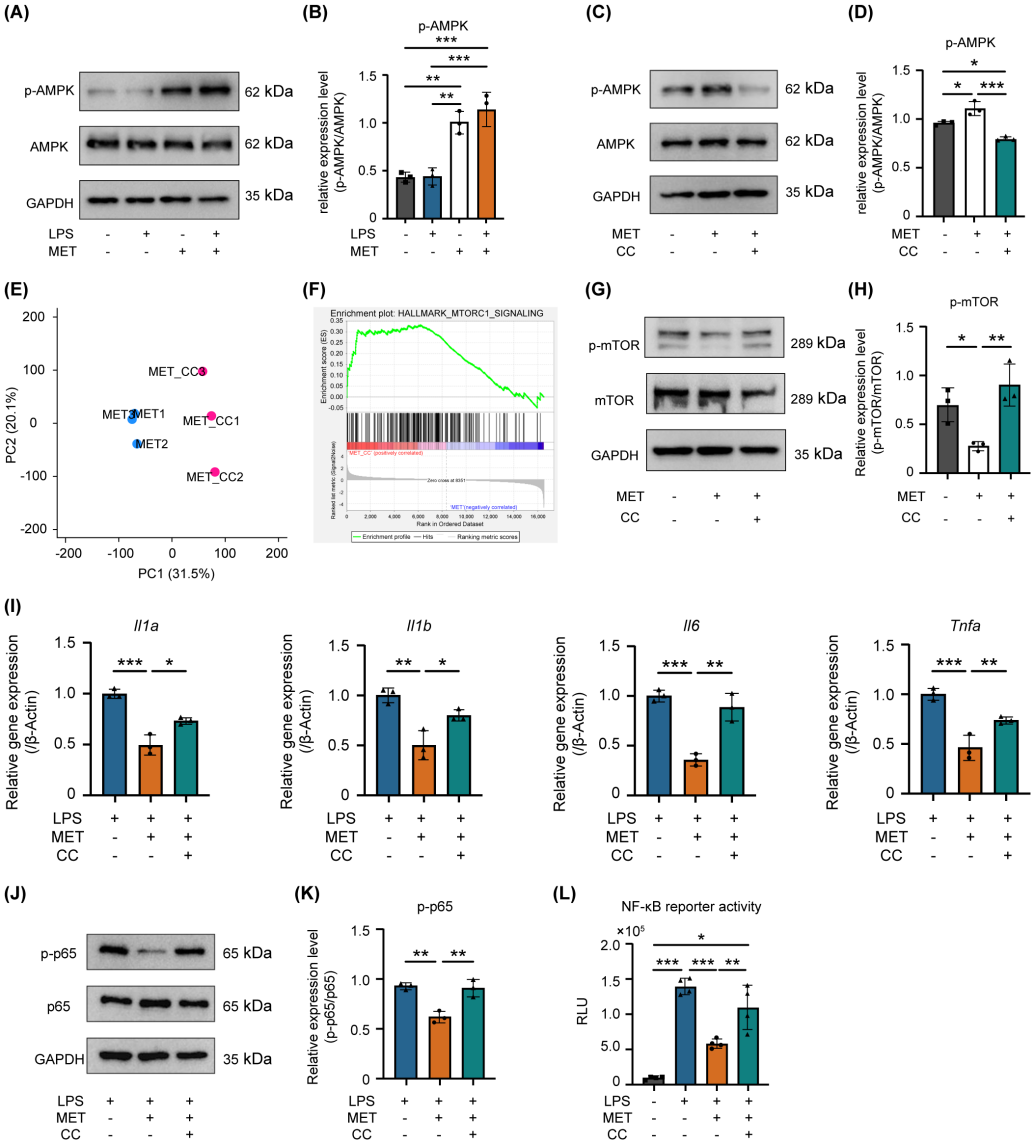
AMPK is involved in MET-mediated suppression of mTOR/NF-κB signaling in LPS-stimulated RAW264.7 cells. **(A, B)** Effect of MET on phosphorylated-AMPK (p-AMPK). Representative western blotting image **(A)**. Quantitative analysis of protein expression level **(B)**. MET enhances the phosphorylation levels of AMPK in RAW264.7 cells, while LPS has no impact on p-AMPK. MET markedly increased the ratio of p-AMPK/AMPK. **(C, D)** Effect of Compound C (CC, 5 μM for 1 h) on MET-induced p-AMPK expression. **(C)** Representative western blot images. Quantitative analysis of protein expression level **(D)**. MET inhibited p-AMPK expression in the presence of the AMPK inhibitor, CC. The ratio of p-AMPK/AMPK was significantly upregulated by MET, which was markedly reduced by CC. **(E)** Gene expression patterns by principal component analysis. MET: metformin-treated RAW264.7 cells. MET+CC: MET and CC co-treated RAW264.7 cells. The expression patterns in MET+CC differed from those in MET. **(F)** Gene set enrichment analysis between MET-treated RAW264.7 cells and MET and CC co-treated RAW264.7 cells. (n = 3). Gene set related to mTOR signaling was upregulated by CC treatment in MET-treated RAW264.7 cells. **(G, H)** Countervailing effect of CC on MET-induced mTOR signaling suppression. A representative image of western blotting **(G)**. Quantitative analysis of protein expression level **(H)**. MET decreased the p-mTOR protein expression level, which returned to the control level in the presence of CC. The ratio of p-mTOR/mTOR is significantly decreased by MET, which was countervailed in the presence of CC. **(I)** Impact of CC on pro-inflammatory cytokine expression in RAW264.7 cells treated with LPS and MET. MET treatment notably reduced LPS-induced pro-inflammatory cytokine expression (*Il1a, Il1b, Il6*, and *Tnfa*), whereas in the presence of CC, the expression levels of these cytokines did not differ from those of the control. **(J, K)** Effect of CC on p-p65 protein expression levels reduced by MET in LPS-treated RAW264.7 cells. Representative western blotting image **(J)**. Quantitative analysis of protein expression levels **(K)**. MET treatment decreased the LPS-induced upregulation of p-p65 expression, which was counteracted in the presence of CC. **(L)** Effect of CC on NF-κB signaling activity inhibited by MET in LPS-treated RAW264.7 cells. A luciferase assay confirmed that CC significantly reduced the inhibitory effect of MET on LPS-induced NF-κB signaling activation (n = 4). Values are shown as the mean ± standard deviation. *P < 0.05, **P < 0.01, and ***P < 0.001; n = 3.

**Figure 6:**
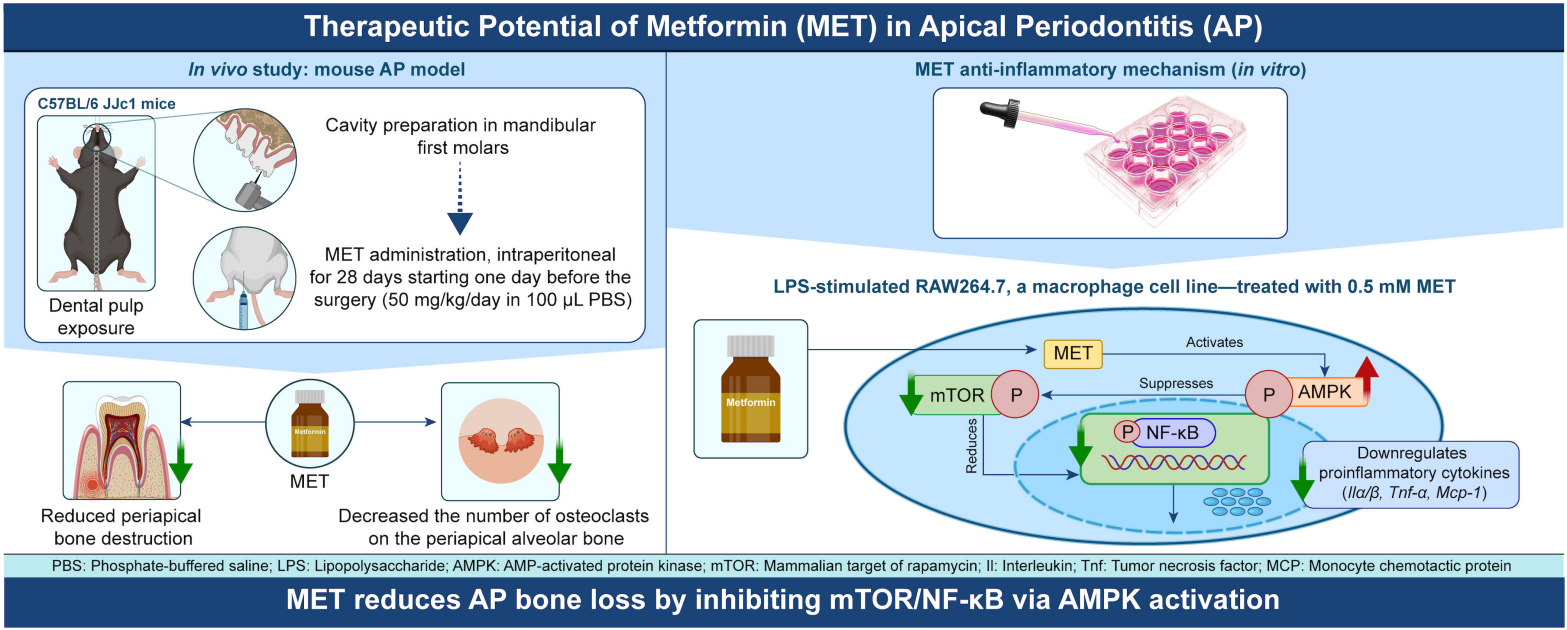
Schematic diagram illustrating the mechanism of anti-inflammatory action of MET during the development of apical periodontitis. MET is transported to macrophages through an organic cation transporter and activates the AMPK signaling pathway. The activation of AMPK signaling suppresses the mTOR signaling pathway, and consequently, NF-κB activity. Inhibiting NF-κB activity reduces the synthesis of inflammatory cytokines such as *Il1a, Il1b, Il6, Tnfa*, and *Mcp1*, which increases during inflammation, thereby reducing inflammatory bone resorption in apical periodontitis. In the periapical region, the reduction in cytokine levels by MET may prevent excessive osteoclast differentiation and activity, thereby mitigating inflammatory bone loss during apical periodontitis. The scheme was created using Adobe Illustrator 2024 and Photoshop 2024 software.

The precise mechanism by which MET downregulates mTOR-mediated NF-κB activation remains unclear. Given that MET activates AMPK signaling by inhibiting mitochondrial complex I (27), the mechanism by which MET-induced AMPK activation modulates mTOR signaling in RAW264.7 cells was examined. Treating these cells with MET notably enhanced AMPK phosphorylation, and this effect was not influenced by LPS stimulation (**Figures 5A, B**). MET-induced AMPK activation was markedly suppressed in the presence of compound C (CC, 5 μM), an AMPK inhibitor (**Figures 5C, D**). Next-generation sequencing analysis comparing MET-treated RAW264.7 cells and those treated with MET and CC combination revealed distinct gene expression profiles between the two groups (the PC1 accounted for 31.5% of the total variance, **Figure 5E**). Notably, gene set enrichment analysis showed the upregulation of mTORC1 signaling in the MET and CC co-treated group compared with that in the MET-treated group (**Figure 5F** and **Table 3**), suggesting that MET-mediated suppression of mTORC1 signaling was attenuated by CC. Western blotting confirmed that CC treatment restored p-mTOR expression that was suppressed by MET (**Figures 5G, H**). In addition, CC attenuated the MET-induced suppression of mRNA expression of pro-inflammatory cytokines *Il1a, Il1b, IL6*, and *Tnfa* in LPS-stimulated RAW264.7 cells (**Figure 5I**). CC also counteracted the inhibitory effects of MET on NF-κB signaling in LPS-stimulated RAW264.7 cells (**Figures 5J, K**). Similarly, CC markedly downregulated the inhibitory effect of MET on LPS-induced NF-κB signaling (**Figure 5L**). These results indicate that MET-induced AMPK activation suppresses NF-κB signaling by inhibiting the mTOR pathway, exerting an anti-inflammatory effect that may contribute to attenuating periapical bone destruction (**Figure 6**).

**Table 3:** Gene set enrichment analysis between MET-treated and combination of MET and CC-treated samples.

Gene set	Size	NES	NOM *P*-Value	FDR q-Value
Up-regulated after CC treatment				
Cholesterol homeostasis	63	1.84	< 0.001	0.008
Tnfα signaling via NF-κB	162	1.66	< 0.001	0.026
Oxidative phosphorylation	184	1.49	< 0.001	0.075
Hypoxia	145	1.48	0.005	0.065
mTORC1 signaling	189	1.39	0.006	0.099
Myc targets v1	189	1.37	0.007	0.096

FDR, false discovery rate; NES, normalized enrichment score; NOM, nominal.

## 4 Discussion

In this study, we demonstrated that the systemic administration of MET significantly inhibited the development of experimentally induced AP. *In vitro* experiments showed that the downregulation of pro-inflammatory cytokines through the AMPK/mTOR axis in macrophages is a crucial mechanism underlying MET-induced AP suppression.

The favorable effects of MET on chronic oral inflammatory diseases have been evaluated in previous studies. Oral administration of MET inhibits alveolar bone resorption caused by a ligature-induced experimental periodontitis model in mice (28). Intracanal application of MET reduces bone resorption in the periapical area in experimentally induced AP models in rodents (29, 30). In addition, intramuscular injections of MET decrease periapical bone loss 28 days after pulp exposure in rats (31). Various mechanisms have been proposed, explaining the reduction in bone loss and the anti-inflammatory effects of MET, including autophagy (28), regulated cell death (29), and modulation of bone metabolism (31). For example, MET activates autophagy through the mTOR pathway and regulates the release of high mobility group box-1 protein induced by LPS (28). MET inhibits cell necroptosis and promotes mitochondrial apoptosis by inhibiting Z-DNA binding protein 1 activation, alleviating inflammation, and promoting bone healing in AP (29). MET has also been shown to enhance the expression of runt-related transcription factor 2 and downregulate the number of TRAP-positive cells in AP (19). Moreover, MET reduces inducible nitric oxide synthase and C-C motif ligand 2 expression, which may contribute to the downregulation of monocyte/macrophage infiltration and activity in AP (30). Emerging evidence suggests that MET retains its anti-inflammatory and bone-protective properties even under hyperglycemic conditions. Systemic administration of MET in diabetic-obese mice has been shown to attenuate periodontal inflammation and inhibit alveolar bone pyroptosis mediated by the Nod-like receptor pyrin domain containing 3 (32). Similarly, delivery of MET via poly (lactic-co-glycolic acid) nanoparticles, effectively reduced ligature-induced alveolar bone resorption in a periodontitis model using type 2 diabetic rat (33). Although these studies focused on periodontal inflammation, the underlying mechanisms are most probably implicated in AP. Collectively, these findings suggest that the therapeutic effects of metformin are preserved, and potentially beneficial, even in hyperglycemic environments. In addition to its immunomodulatory effects, MET may also influence osteoclast differentiation and activity. In the present study, a reduction in TRAP-positive cells was observed *in vivo*, which could reflect effects on both inflammatory signaling and osteoclast-lineage cells. Supporting this possibility, MET has been shown to suppress RANKL-induced osteoclastogenesis through AMPK-dependent mechanisms (34), and to attenuate osteoclast-mediated bone resorption *in vivo* via the AMPK/NF-κB/ERK signaling pathway (35). Although the current work primarily focused on the mechanism of the anti-inflammatory effect, these findings suggest that the anti-resorptive effects of MET may involve multiple, complementary mechanisms, including modulation of both immune responses and osteoclastogenesis. However, the precise mechanisms by which MET exerts its effects require further clarification.

*In vitro* experiments using RAW264.7 cells, a macrophage cell line (36), revealed that MET treatment alleviated LPS-induced pro-inflammatory cytokine expression. Furthermore, AMPK signaling is the principal pathway activated by MET. MET is transported into cells through organic cation transport proteins and primarily inhibits mitochondrial complex I, leading to an increased AMP/ATP ratio and subsequent AMPK activation (37–39). AMPK activation regulates immune-mediated inflammation in various immune cells. For instance, MET promotes the differentiation and activation of CD4+ and CD8+ T lymphocytes through the AMPK/mTORC1 pathway (40). Notably, MET enhances neutrophil chemotaxis and bacterial uptake independent of the mTORC1 pathway (41). In addition, it inhibits the polarization of monocytes into M1 macrophages and decreases the production of reactive oxygen species (42).

In the present study, the findings show that AMPK activation by MET downregulates mTORC1 signaling, suggesting that MET exerts its anti-inflammatory effects through the AMPK-mTOR axis in macrophages. The mTOR signaling pathway plays a key role in regulating NF-κB signaling, as it promotes the phosphorylation of p65 (26), a critical step in NF-κB activation. NF-κB signaling is widely recognized as a pivotal pathway in mediating inflammatory responses, driving the expression of pro-inflammatory cytokines, chemokines, and adhesion molecules (43, 44). Reportedly, activating the mTOR-NF-κB axis is a key pathway in exacerbating vascular inflammation (45). In addition, under high glucose conditions, mTOR facilitates the translocation of NF-κB to the nucleus, promoting the production of pro-inflammatory cytokines in hippocampus neurons, highlighting the role of the mTOR/NF-κB pathway in diabetic encephalopathy (46). Findings from this study indicated that MET inhibited mTOR-dependent NF-κB signaling through AMPK activation in macrophages. Suppressing the mTOR-NF-κB axis by MET may reduce the expression of pro-inflammatory mediators, potentially contributing to inflammation attenuation and subsequent suppression of AP development.

However, the precise mechanisms underlying MET-mediated immune regulation remain incompletely understood. While the present study emphasizes the AMPK–mTOR–NF-κB axis, several AMPK-independent pathways have also been reported (47). For instance, MET has been shown to reduce activation of the NOD-like receptor family pyrin domain-containing three inflammasome and IL-1β secretion by inhibiting mitochondrial ATP and DNA synthesis in bone marrow-derived macrophages (48). MET also suppresses the fatty acid synthase/protein kinase B pathway and its downstream mitogen-activated protein kinase signaling, which may contribute to the reduction of inflammatory responses (17). Additionally, MET may inhibit monocyte-to-macrophage differentiation by downregulating the phosphorylation of signal transducer and activator of transcription 3 (49). These findings support the idea that MET regulates macrophage activity and cytokine expression through multiple intracellular signaling cascades beyond AMPK, highlighting the complexity of its immunomodulatory functions. These findings show that there could be additional, as yet unidentified, mechanisms through which MET exerts its immunomodulatory effects. Furthermore, bone resorption in AP results from the accumulation and activation of osteoclasts, which is promoted by chronic inflammation modulated by infiltrated immune cells (50). A comprehensive understanding of MET-mediated immune modulation, achieved by combining histological and bioinformatics analyses across different stages of AP progression, could provide novel insights and therapeutic approaches for AP treatment.

This study has several limitations. Although H&E staining demonstrated reduced inflammatory cell infiltration in the MET-treated group, it does not allow for precise identification of macrophage phenotypes or the expression levels of inflammatory cytokines. A previous study, in which MET was used as an intracanal medication during root canal treatment (30), reported a reduction in iNOS- and CD68-positive cells within periapical lesions, supporting the M1-suppressive effect of MET. Since bone resorption is driven by prolonged inflammation involving immune cells and osteoclasts, it is possible that key cellular and molecular events in the early phase of AP were not fully captured. In addition, the lack of quantitative analysis of cytokine expression and immune cell distribution in the periapical lesions limits the ability to establish translational relevance between the *in vivo* and *in vitro* findings in the present study. Future research could be enhanced by time-course analyses that incorporate histological evaluation and transcriptomic profiling, in order to gain deeper understanding of the temporal dynamics of immune responses and osteoclastic activity, and to further clarify the mechanisms by which MET modulates inflammation in AP. Only male mice were used in this study to minimize biological variability caused by hormonal fluctuations in females, which are known to influence immune responses and drug sensitivity (51, 52). In addition, most established experimental models of AP are based on male mice, allowing for methodological consistency and improved reproducibility (53). While this approach enhances internal validity, it may limit the generalizability of the findings. Future studies including both sexes will be necessary to investigate potential sex-specific differences in the response to MET.

In conclusion, the findings from this study show that MET effectively attenuates periapical bone destruction in experimentally induced AP and that MET-induced activation of AMPK inhibited mTOR-dependent NF-κB signaling, resulting in reduced pro-inflammatory cytokine expression in RAW264.7 macrophages stimulated with LPS.

## Data Availability

The data presented in the study are deposited in the NCBI BioProject repository, accession number PRJNA1253073.

## Supplementary material

Supplementary Figure 1
**(A)** Changes in experimentally induced periapical lesion size in the groups treated with MET or PBS. In the PBS-control group, periapical bone loss was observed from postoperative day (POD) 7 with remarkable bone loss at POD 21 and 28. In contrast, periapical bone destruction progressed from POD 14, and extensive bone loss was not observed at POD 21 and 28 in the MET group. Statistical significance among PBS groups and MET group are indicated by uppercase letters **(A-C)**, and lowercase letters (a-c), respectively. Different letters denote statistically significant differences (n = 6, *P* <.05). Values are shown as the mean ± standard deviation.

Supplementary Figure 2
**(A, B)** Phosphorylation of p65 in response to LPS stimulation. The western blot analysis was repeated thrice, and a representative image is shown **(A)**. The expression of phosphorylated-p65 (p-p65) peaked at 30 min after LPS stimulation (**B**, n = 3). Thus, RAW264.7 cells were treated with 100 ng/mL LPS for 30 min to stimulate the phosphorylation of p65 in later studies. **(C)** Effect of LPS on NF-κB activation. Luciferase assay confirmed that LPS treatment promotes NF-κB activity, which peaked at 4 h (n = 4). Values are shown as the mean ± standard deviation. **P* < .05, ***P* < .001, and *****P* < .0001.

Supplementary Figure 3
**(A)** mRNA expression of pro-inflammatory cytokines in the presence of LPS, BAY11-7085, and MHY in RAW264.7 cells. BAY 11-7085, an NF-κB inhibitor blocks the upregulation of the expression of pro-inflammatory cytokines (*Il1a, Il1b*, and *Mcp1*) by LPS treatment. Repressive effect of BAY on mRNA expression of Il1a and Il1b was impaired in the presence of MHY (mTOR agonist). **(B)** Effect of MHY on NF-κB activation impaired by BAY 11–7085 in LPS-treated RAW264.7 cells. Luciferase assay confirmed that LPS treatment promoted NF-κB activity, which was suppressed by BAY 11-7085. However, in the presence of MHY, the NF-κB activity was reupregulated, suggesting that mTOR signaling alone promotes NF-κB activation. Values are shown as the mean ± standard deviation. **P* < .05, ***P* < .001, *****P* < .0001; n = 3.

Supplementary Figure 4
**(A, B)** Effect of various concentrations of compound C (CC) on phosphorated-AMPK expression. A representative image of western blotting **(A)**. Quantitative analysis of protein expression level **(B)** CC at a concentration > 5 mM effectively decreases phosphorated-AMPK expression levels. Values are shown as the mean ± standard deviation. ***P* < .001; n = 3.

## References

[1] Marton IJ Kiss C. Protective and destructive immune reactions in apical periodontitis. Oral Microbiol Immunol. (2000) 15:139–50. doi: 10.1034/j.1399-302x.2000.150301.x, PMID: 11154396

[2] Nair PN. Apical periodontitis: A dynamic encounter between root canal infection and host response. Periodontol 2000. (1997) 13:121–48. doi: 10.1111/j.1600-0757.1997.tb00098.x, PMID: 9567926

[3] Stashenko P Teles R D’Souza R. Periapical inflammatory responses and their modulation. Crit Rev Oral Biol Med. (1998) 9:498–521. doi: 10.1177/10454411980090040701, PMID: 9825224

[4] West J. Endodontic update 2006. J Esthet Restor Dent. (2006) 18:280–300. doi: 10.1111/j.1708-8240.2006.00039.x, PMID: 16987326

[5] Wen YH Lin YX Zhou L Lin C Zhang L. The immune landscape in apical periodontitis: from mechanism to therapy. Int Endod J. (2024) 57:1526–45. doi: 10.1111/iej.14125, PMID: 39087849

[6] Kawashima N Okiji T Kosaka T Suda H. Kinetics of macrophages and lymphoid cells during the development of experimentally induced periapical lesions in rat molars: A quantitative immunohistochemical study. J Endod. (1996) 22:311–6. doi: 10.1016/S0099-2399(96)80266-4, PMID: 8934992

[7] Franca GM Carmo AFD Costa Neto H Andrade A Lima KC Galvao HC. Macrophages subpopulations in chronic periapical lesions according to clinical and morphological aspects. Braz Oral Res. (2019) 33:e047. doi: 10.1590/1807-3107bor-2019.vol33.0047, PMID: 31141038

[8] Braz-Silva PH Bergamini ML Mardegan AP De Rosa CS Hasseus B Jonasson P. Inflammatory profile of chronic apical periodontitis: A literature review. Acta Odontol Scand. (2019) 77:173–80. doi: 10.1080/00016357.2018.1521005, PMID: 30585523

[9] Song Y Li X Huang D Song H. Macrophages in periapical lesions: potential roles and future directions. Front Immunol. (2022) 13:949102. doi: 10.3389/fimmu.2022.949102, PMID: 36131939 PMC9483141

[10] Kawashima N Stashenko P. Expression of bone-resorptive and regulatory cytokines in murine periapical inflammation. Arch Oral Biol. (1999) 44:55–66. doi: 10.1016/s0003-9969(98)00094-6, PMID: 10075151

[11] Wang CY Tani-Ishii N Stashenko P. Bone-resorptive cytokine gene expression in periapical lesions in the rat. Oral Microbiol Immunol. (1997) 12:65–71. doi: 10.1111/j.1399-302x.1997.tb00619.x, PMID: 9227128

[12] Dal-Fabbro R Yu M Mei L Sasaki H Schwendeman A Bottino MC. Synthetic high-density lipoprotein (Shdl): A bioinspired nanotherapeutics for managing periapical bone inflammation. Int J Oral Sci. (2024) 16:50. doi: 10.1038/s41368-024-00316-w, PMID: 38956025 PMC11219839

[13] Shapouri-Moghaddam A Mohammadian S Vazini H Taghadosi M Esmaeili SA Mardani F. Macrophage plasticity, polarization, and function in health and disease. J Cell Physiol. (2018) 233:6425–40. doi: 10.1002/jcp.26429, PMID: 29319160 PMC13482560

[14] Song W Ye L Tang Q Lu X Huang X Xie M. Rev-erbalpha attenuates refractory periapical periodontitis via M1 polarization: an *in vitro* and *in vivo* study. Int Endod J. (2024) 57:451–63. doi: 10.1111/iej.14024, PMID: 38279698

[15] Viollet B Guigas B Sanz Garcia N Leclerc J Foretz M Andreelli F. Cellular and molecular mechanisms of metformin: an overview. Clin Sci (Lond). (2012) 122:253–70. doi: 10.1042/CS20110386, PMID: 22117616 PMC3398862

[16] Isoda K Young JL Zirlik A MacFarlane LA Tsuboi N Gerdes N. Metformin inhibits proinflammatory responses and nuclear factor-kappab in human vascular wall cells. Arterioscler Thromb Vasc Biol. (2006) 26:611–7. doi: 10.1161/01.ATV.0000201938.78044.75, PMID: 16385087

[17] Xiong W Sun KY Zhu Y Zhang X Zhou YH Zou X. Metformin alleviates inflammation through suppressing fasn-dependent palmitoylation of akt. Cell Death Dis. (2021) 12:934. doi: 10.1038/s41419-021-04235-0, PMID: 34642298 PMC8511025

[18] Ren C Hao X Wang L Hu Y Meng L Zheng S. Metformin carbon dots for promoting periodontal bone regeneration via activation of erk/ampk pathway. Adv Healthc Mater. (2021) 10:e2100196. doi: 10.1002/adhm.202100196, PMID: 33987977

[19] Hong CY Lin SK Wang HW Shun CT Yang CN Lai EH. Metformin reduces bone resorption in apical periodontitis through regulation of osteoblast and osteoclast differentiation. J Endod. (2023) 49:1129–37. doi: 10.1016/j.joen.2023.07.005, PMID: 37454872

[20] Tazawa K Sasaki H. Three-dimensional cellular visualization in mouse apical periodontitis using combined whole-mount staining and optical tissue clearing. J Oral Biosci. (2023) 65:132–5. doi: 10.1016/j.job.2022.12.003, PMID: 36587735 PMC10299740

[21] LaMoia TE Shulman GI. Cellular and molecular mechanisms of metformin action. Endocr Rev. (2021) 42:77–96. doi: 10.1210/endrev/bnaa023, PMID: 32897388 PMC7846086

[22] Goldman E Reich E Abramovitz I Klutstein M. Inducing apical periodontitis in mice. J Vis Exp. (2019) 150). doi: 10.3791/59521, PMID: 31449241

[23] Alchawoosh A Hashimoto K Kawashima N Noda S Nozaki K Okiji T. Hydraulic calcium silicate-based root canal sealers mitigate proinflammatory cytokine synthesis and promote osteogenesis *in vitro* . J Dent Sci. (2023) 18:1731–9. doi: 10.1016/j.jds.2022.12.019, PMID: 37799856 PMC10547950

[24] Powell JD Pollizzi KN Heikamp EB Horton MR. Regulation of immune responses by mtor. Annu Rev Immunol. (2012) 30:39–68. doi: 10.1146/annurev-immunol-020711-075024, PMID: 22136167 PMC3616892

[25] Lee J Rhee MH Kim E Cho JY. Bay 11–7082 is a broad-spectrum inhibitor with anti-inflammatory activity against multiple targets. Mediators Inflammation. (2012) 2012:416036. doi: 10.1155/2012/416036, PMID: 22745523 PMC3382285

[26] Zhou M Xu W Wang J Yan J Shi Y Zhang C. Boosting mtor-dependent autophagy via upstream tlr4-myd88-mapk signalling and downstream Nf-Kappab pathway quenches intestinal inflammation and oxidative stress injury. EBioMedicine. (2018) 35:345–60. doi: 10.1016/j.ebiom.2018.08.035, PMID: 30170968 PMC6161481

[27] Foretz M Guigas B Bertrand L Pollak M Viollet B. Metformin: from mechanisms of action to therapies. Cell Metab. (2014) 20:953–66. doi: 10.1016/j.cmet.2014.09.018, PMID: 25456737

[28] Sun B Ying S Ma Q Li H Li J Song J. Metformin ameliorates Hmgb1-mediated oxidative stress through mtor pathway in experimental periodontitis. Genes Dis. (2023) 10:542–53. doi: 10.1016/j.gendis.2021.06.003, PMID: 37223504 PMC10201554

[29] Liu H Liu YX Fan W Fan B. Metformin switches cell death modes to soothe the apical periodontitis via Zbp1. FASEB J. (2024) 38:e23549. doi: 10.1096/fj.202302073R, PMID: 38446465

[30] Wang HW Lai EH Yang CN Lin SK Hong CY Yang H. Intracanal metformin promotes healing of apical periodontitis via suppressing inducible nitric oxide synthase expression and monocyte recruitment. J Endod. (2020) 46:65–73. doi: 10.1016/j.joen.2019.10.001, PMID: 31753516

[31] Liu L Zhang C Hu Y Peng B. Protective effect of metformin on periapical lesions in rats by decreasing the ratio of receptor activator of nuclear factor kappa B ligand/osteoprotegerin. J Endod. (2012) 38:943–7. doi: 10.1016/j.joen.2012.03.010, PMID: 22703658

[32] Zhou X Wang Q Nie L Zhang P Zhao P Yuan Q. Metformin ameliorates the Nlpp3 inflammasome mediated pyroptosis by inhibiting the expression of Nek7 in diabetic periodontitis. Arch Oral Biol. (2020) 116:104763. doi: 10.1016/j.archoralbio.2020.104763, PMID: 32480011

[33] Pereira A Brito GAC Lima MLS Silva Junior AAD Silva EDS de Rezende AA. Metformin hydrochloride-loaded plga nanoparticle in periodontal disease experimental model using diabetic rats. Int J Mol Sci. (2018) 19. doi: 10.3390/ijms19113488, PMID: 30404181 PMC6274734

[34] Kim YS Park BS Baek HS Kang HM Oh JM Kim IR. Metformin activates Ampk and mtor to inhibit Rankl-stimulated osteoclast formation. Eur Rev Med Pharmacol Sci. (2023) 27:8795–811. doi: 10.26355/eurrev_202309_33801, PMID: 37782190

[35] Guo H Ding D Wang L Yan J Ma L Jin Q. Metformin attenuates osteoclast-mediated abnormal subchondral bone remodeling and alleviates osteoarthritis via Ampk/Nf-Kappab/Erk signaling pathway. PloS One. (2021) 16:e0261127. doi: 10.1371/journal.pone.0261127, PMID: 34914744 PMC8675877

[36] Kong L Smith W Hao D. Overview of Raw264.7 for osteoclastogensis study: phenotype and stimuli. J Cell Mol Med. (2019) 23:3077–87. doi: 10.1111/jcmm.14277, PMID: 30892789 PMC6484317

[37] Cetin M Sahin S. Microparticulate and nanoparticulate drug delivery systems for metformin hydrochloride. Drug Delivery. (2016) 23:2796–805. doi: 10.3109/10717544.2015.1089957, PMID: 26394019

[38] Boyle JG Salt IP McKay GA. Metformin action on amp-activated protein kinase: A translational research approach to understanding a potential new therapeutic target. Diabetes Med. (2010) 27:1097–106. doi: 10.1111/j.1464-5491.2010.03098.x, PMID: 20854376

[39] Rena G Hardie DG Pearson ER. The mechanisms of action of metformin. Diabetologia. (2017) 60:1577–85. doi: 10.1007/s00125-017-4342-z, PMID: 28776086 PMC5552828

[40] Nojima I Wada J. Metformin and its immune-mediated effects in various diseases. Int J Mol Sci. (2023) 24. doi: 10.3390/ijms24010755, PMID: 36614197 PMC9821749

[41] Park DW Jiang S Tadie JM Stigler WS Gao Y Deshane J. Activation of Ampk enhances neutrophil chemotaxis and bacterial killing. Mol Med. (2013) 19:387–98. doi: 10.2119/molmed.2013.00065, PMID: 24091934 PMC3883969

[42] Nassif RM Chalhoub E Chedid P Hurtado-Nedelec M Raya E Dang PM. Metformin inhibits ros production by human M2 macrophages via the activation of ampk. Biomedicines. (2022) 10. doi: 10.3390/biomedicines10020319, PMID: 35203528 PMC8869356

[43] Yu H Lin L Zhang Z Zhang H Hu H. Targeting Nf-Kappab pathway for the therapy of diseases: mechanism and clinical study. Signal Transduct Target Ther. (2020) 5:209. doi: 10.1038/s41392-020-00312-6, PMID: 32958760 PMC7506548

[44] Liu T Zhang L Joo D Sun SC. Nf-Kappab signaling in inflammation. Signal Transduct Target Ther. (2017) 2:17023–. doi: 10.1038/sigtrans.2017.23, PMID: 29158945 PMC5661633

[45] Li Y Yang L Dong L Yang ZW Zhang J Zhang SL. Crosstalk between the Akt/Mtorc1 and Nf-Kappab signaling pathways promotes hypoxia-induced pulmonary hypertension by increasing Dpp4 expression in Pasmcs. Acta Pharmacol Sin. (2019) 40:1322–33. doi: 10.1038/s41401-019-0272-2, PMID: 31316183 PMC6786428

[46] Xu T Liu J Li XR Yu Y Luo X Zheng X. The Mtor/Nf-Kappab pathway mediates neuroinflammation and synaptic plasticity in diabetic encephalopathy. Mol Neurobiol. (2021) 58:3848–62. doi: 10.1007/s12035-021-02390-1, PMID: 33860440

[47] Bone NB Becker EJ Jr. Husain M Jiang S Zmijewska AA Park DW. Ampk activates parkin independent autophagy and improves post sepsis immune defense against secondary bacterial lung infections. Sci Rep. (2021) 11:12387. doi: 10.1038/s41598-021-90573-0, PMID: 34117280 PMC8196038

[48] Xian H Liu Y Rundberg Nilsson A Gatchalian R Crother TR Tourtellotte WG. Metformin inhibition of mitochondrial Atp and DNA synthesis abrogates Nlrp3 inflammasome activation and pulmonary inflammation. Immunity. (2021) 54:1463–77 e11. doi: 10.1016/j.immuni.2021.05.004, PMID: 34115964 PMC8189765

[49] Vasamsetti SB Karnewar S Kanugula AK Thatipalli AR Kumar JM Kotamraju S. Metformin inhibits monocyte-to-macrophage differentiation via Ampk-mediated inhibition of Stat3 activation: potential role in atherosclerosis. Diabetes. (2015) 64:2028–41. doi: 10.2337/db14-1225, PMID: 25552600

[50] Takahashi K. Microbiological, pathological, inflammatory, immunological and molecular biological aspects of periradicular disease. Int Endod J. (1998) 31:311–25. doi: 10.1046/j.1365-2591.1998.00171.x, PMID: 9823133

[51] Harraghy N Regamey A Girod PA Mermod N. Identification of a potent Mar element from the mouse genome and assessment of its activity in stable and transient transfections. J Biotechnol. (2011) 154:11–20. doi: 10.1016/j.jbiotec.2011.04.004, PMID: 21540066

[52] Mackey DA Kearns LS Hewitt AW. Gene-based therapies for leber hereditary optic neuropathy. Hype Hope? Asia Pac J Ophthalmol (Phila). (2016) 5:253–5. doi: 10.1097/APO.0000000000000220, PMID: 27488066

[53] Yang F Zhang Y Chen Z Zhang L. Vista blockade aggravates bone loss in experimental murine apical periodontitis. Front Immunol. (2021) 12:738586. doi: 10.3389/fimmu.2021.738586, PMID: 34691045 PMC8529274

