# Supplementary figures and images for: Metformin attenuates alveolar bone destruction in mice with apical periodontitis and inhibits pro-inflammatory cytokine synthesis in lipopolysaccharide-stimulated RAW264.7 through the AMPK-mTOR-NF-κB pathway
Source: Front Immunol. 2025 Jul 31;16:1643676. doi: 10.3389/fimmu.2025.1643676 (PMC12351400; doi:10.3389/fimmu.2025.1643676)

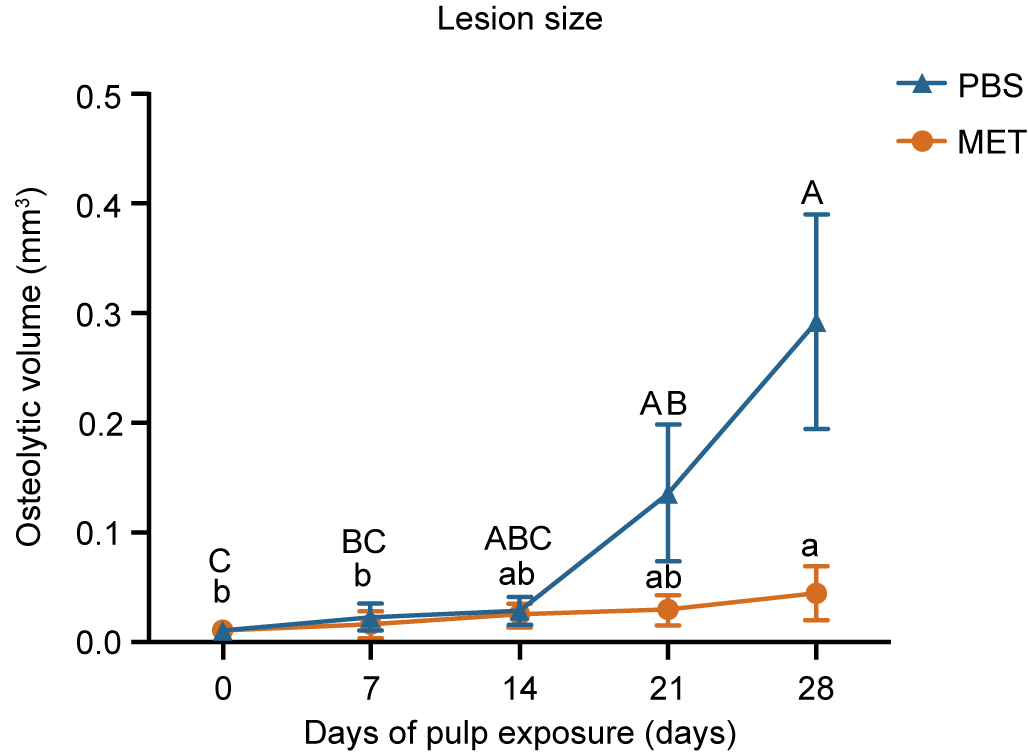

Supplement: Supplementary Figure 1 — (A) Changes in experimentally induced periapical lesion size in the groups treated with MET or PBS. In the PBS-control group, periapical bone loss was observed from postoperative day (POD) 7 with remarkable bone loss at POD 21 and 28. In contrast, periapical bone destruction progressed from POD 14, and extensive bone loss was not observed at POD 21 and 28 in the MET group. Statistical significance among PBS groups and MET group are indicated by uppercase letters (A-C), and lowercase letters (a-c), respectively. Different letters denote statistically significant differences (n = 6, P <.05). Values are shown as the mean ± standard deviation. [file Image1.tif]

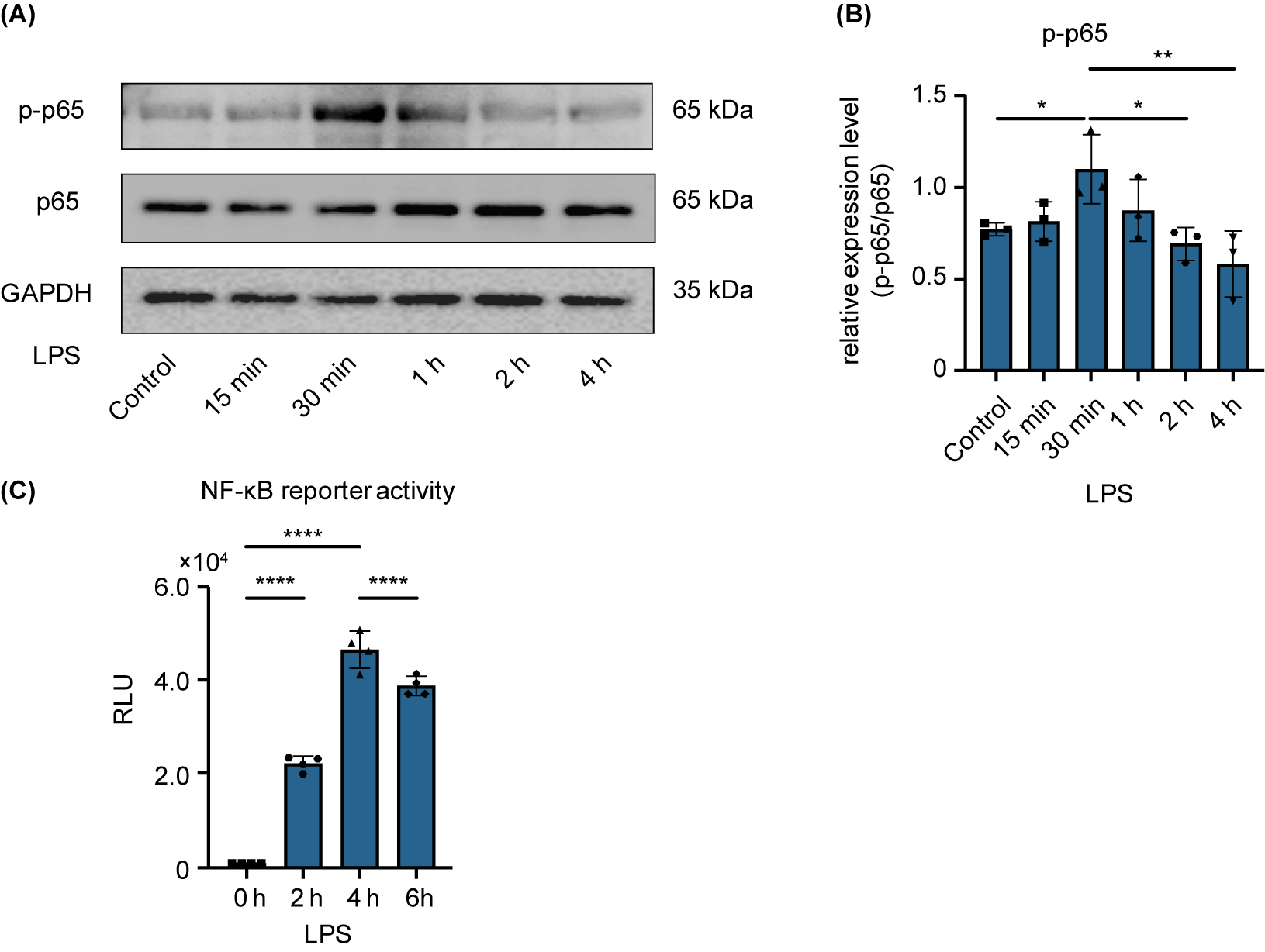

Supplement: Supplementary Figure 2 — (A, B) Phosphorylation of p65 in response to LPS stimulation. The western blot analysis was repeated thrice, and a representative image is shown (A). The expression of phosphorylated-p65 (p-p65) peaked at 30 min after LPS stimulation (B, n = 3). Thus, RAW264.7 cells were treated with 100 ng/mL LPS for 30 min to stimulate the phosphorylation of p65 in later studies. (C) Effect of LPS on NF-κB activation. Luciferase assay confirmed that LPS treatment promotes NF-κB activity, which peaked at 4 h (n = 4). Values are shown as the mean ± standard deviation. *P < .05, **P < .001, and ****P < .0001. [file Image2.tif]

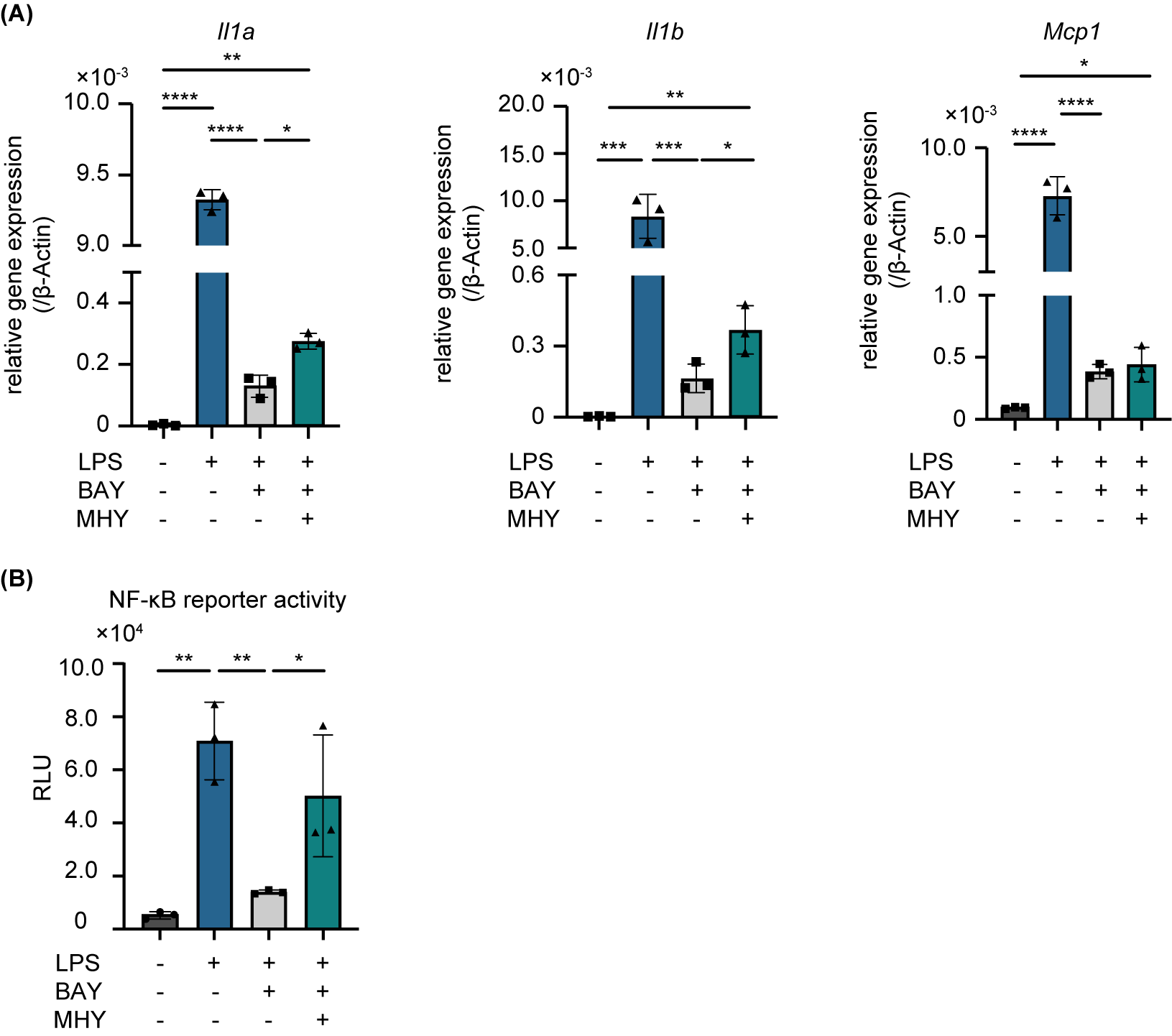

Supplement: Supplementary Figure 3 — (A) mRNA expression of pro-inflammatory cytokines in the presence of LPS, BAY11-7085, and MHY in RAW264.7 cells. BAY 11-7085, an NF-κB inhibitor blocks the upregulation of the expression of pro-inflammatory cytokines (Il1a, Il1b, and Mcp1) by LPS treatment. Repressive effect of BAY on mRNA expression of Il1a and Il1b was impaired in the presence of MHY (mTOR agonist). (B) Effect of MHY on NF-κB activation impaired by BAY 11–7085 in LPS-treated RAW264.7 cells. Luciferase assay confirmed that LPS treatment promoted NF-κB activity, which was suppressed by BAY 11-7085. However, in the presence of MHY, the NF-κB activity was reupregulated, suggesting that mTOR signaling alone promotes NF-κB activation. Values are shown as the mean ± standard deviation. *P < .05, **P < .001, ****P < .0001; n = 3. [file Image3.tif]

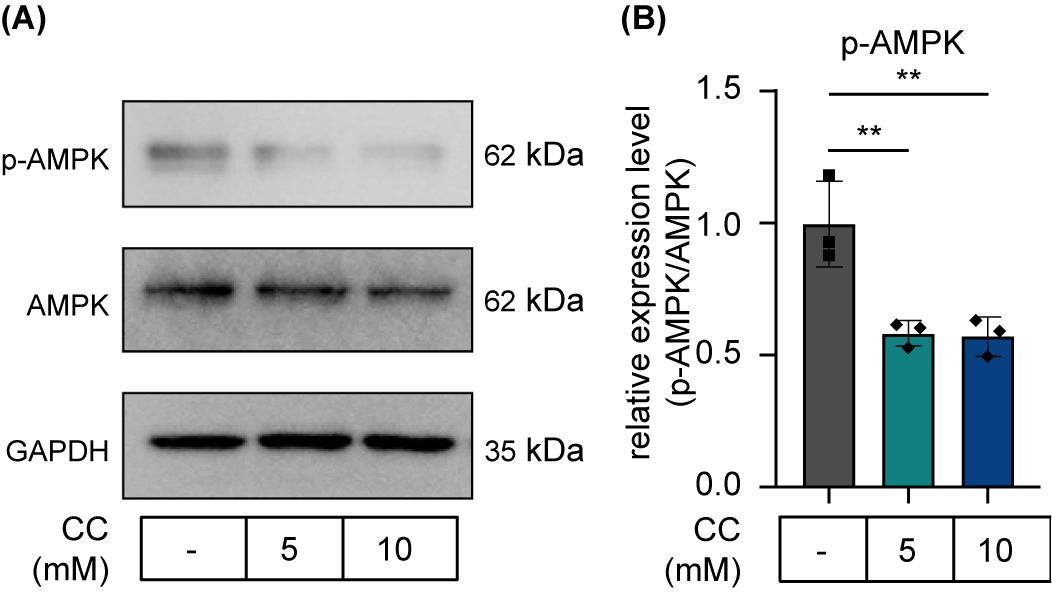

Supplement: Supplementary Figure 4 — (A, B) Effect of various concentrations of compound C (CC) on phosphorated-AMPK expression. A representative image of western blotting (A). Quantitative analysis of protein expression level (B) CC at a concentration > 5 mM effectively decreases phosphorated-AMPK expression levels. Values are shown as the mean ± standard deviation. **P < .001; n = 3. [file Image4.tif]
